# The Effectiveness of Preventative Interventions to Reduce Mental Health Problems in at-risk Children and Young People: A Systematic Review of Reviews

**DOI:** 10.1007/s10935-024-00785-z

**Published:** 2024-06-17

**Authors:** R. McGovern, A. Balogun-Katung, B. Artis, B. Bareham, L. Spencer, H. Alderson, E. Brown, J. Brown, R. Lingam, P. McArdle, J. J. Newham, A. Wojciechowska, J. Rankin, S. Redgate, P. Thomason, E. Kaner

**Affiliations:** 1 Population Health Sciences Institute, Newcastle University, Baddiley-Clark Building, Richardson Road, Newcastle upon Tyne, NE2 4AX UK; 2 Children’s Social Care, Newcastle City Council, Newcastle upon Tyne, UK; 3 Population Child Health Research Group, School of Women and Children’s Health, University New South Wales, Sydney, Australia; 4 Child and Adolescent Mental Health Services, Cumbria, Northumberland, Tyne and Wear NHS Foundation Trust, Newcastle upon Tyne, UK; 5 Faculty of Health and Life Sciences, Northumbria University, Newcastle upon Tyne, UK; 6 Disabilities Integrated Team at the Tees, Esk and Wear Valleys, NHS Foundation Trust, Newcastle upon Tyne, UK; 7 Children’s Social Care, Gateshead Council, Tyne and Wear, UK

**Keywords:** Mental health, Child and adolescent, Secondary prevention, Systematic review

## Abstract

Mental health problems are the leading cause of childhood disability worldwide, resulting in poor outcomes for children and young people that persist into adulthood. It is essential that those young people most at risk of developing mental health problems receive effective preventative interventions. Whilst there have been a number of systematic reviews which have examined the effectiveness of secondary prevention interventions for specific groups of children and young people, or to address identified mental health concerns, no review has engaged with the breadth of this literature. We conducted a systematic review of systematic reviews to map this complex field of secondary preventative interventions and identify effective interventions to prevent mental health problems in children and adolescents aged 3–17 years. The review protocol was registered on PROSPERO. We searched five electronic databases from inception to February 2023. The certainty of the evidence was appraised using the AMSTAR 2. We included 49 unique systematic reviews each including between 2 and 249 (mean 34) unique studies; the majority of which were reviews which included only or mostly randomised controlled trials (70%). The reviews examined selective interventions (defined as interventions which are delivered to sub-group populations of young people at increased risk of mental health problems) (n = 22), indicated interventions (defined as interventions which target young people who are found to have pre-clinical symptoms) (n = 15) or a synthesis of both (n = 12). The certainty of the evidence in the reviews was rated as high, (n = 12) moderate (n = 5), low (n = 9) and critically low (n = 23). We found evidence to support both selective and indicated interventions in a range of populations and settings, with most of this evidence available for children and young people in their mid-years (6–10 years) and early adolescence (11–13 years). There was a large body of evidence suggesting that resilience enhancing, cognitive behaviour therapy-based and psychoeducational interventions for children who experience adversity, or those with subclinical externalising problems may offer promise. Early selective interventions for a subpopulation of children and young people who have experienced adversity which combines risk reduction and resilience enhancing approaches directed at children and their families may be effective at reducing mental health problems.

**Supplementary Information:**

The online version contains supplementary material available at 10.1007/s10935-024-00785-z.

## Introduction

Mental health problems are the leading contributor to childhood disease burden worldwide (Baranne & Falissard, 2018; Erskine et al., 2015). Half of all adult mental health disorders emerge before the age of 14 years and three-quarters before the age of 24 years (Kessler et al., 2005). In the UK it is estimated that one in six children aged 5–16 years have a mental health problem (Digital, 2020). These children also experience poor outcomes including an increased risk of substance use (Hopfer et al., 2013), involvement in offending behaviour (Rijo et al., 2016), difficulties in their relationships with family and friends (Lim et al., 2020), diminished educational opportunities, Wickersham et al. (2021) and unplanned pregnancy/parenthood (Clayborne et al., 2019). Children who experience mental health problems are also at risk of persistent mental health problems in adulthood (Wykes et al., 2023) and later economic adversity (Evensen et al., 2017).

The rising cases of adolescent mental health problems over recent years (Wykes et al., 2023), combined with the clinically elevated rates of adolescent depression and anxiety following the COVID-19 pandemic (Racine et al., 2021), are of substantial public health concern. Whilst there are an increasing number of evidence-based interventions to treat mental health problems in children and young people (Pennant et al., 2015; Reynolds et al., 2012; Tindall et al., 2017), this treatment is not accessible to all young people (Department of Health, 2015; Radez et al., 2021), and those who do access treatment often experience long waiting times (Fargas-Malet & McSherry, 2018; Department of Health, 2015). To bring about immediate psychological and social benefits for children, young people, and families whilst also decreasing the prevalence of mental health disorders in adults in the future, effective prevention is required (Wykes et al., 2023).

There is robust evidence for primary prevention of mental health problems. Such interventions are delivered universally to children and young people and have been found to promote positive mental health and prevent the onset of mental health disorders (World Health Organization, 2020). However, it is likely that children who experience greater risk of developing mental health problems may need a more targeted approach which is proportionate to their needs (Marmot et al., 2010). There is a large and diverse evidence-base examining the effectiveness of secondary preventive interventions delivered to children and young people at greater risk than the general population. These interventions which seek to reduce the prevalence of mental health disorders in a population (Institute of Medicine Committee on Prevention of Mental, 1994) may be either selective or indicated. Selective interventions target individuals who are at increased risk of developing mental health disorders on the basis of biological, psychological or social risk factors, whereas indicated interventions target individuals who are identified as having pre-clinical symptoms (Cho & Shin, 2013). These secondary preventive interventions vary depending upon the age of the child, the nature of mental health risk, and the approach taken to improving mental health outcomes. However, they broadly either seek to strengthen resilience (Dray et al., 2017) or modify risk factors within the context or the young person’s response to them (World Health Organization, 2004) in order to reduce the incidence, prevalence or reoccurrence of mental health problems.

There have been multiple systematic reviews which have estimated the effectiveness of secondary preventative interventions for child and adolescent mental health. This includes reviews of interventions within school settings (Gee et al., 2020; Hugh-Jones et al., 2021; Neil & Christensen, 2009), targeted interventions for young people with sub-threshold behavioural problems (Graaf et al., 2008), depression (Bertha & Balazs, 2013) and anxiety (Hugh-Jones et al., 2021), and of selective interventions with subgroups of young people who are considered at elevated risk due to adversity (Havinga et al., 2021), physical health problems (Thabrew et al., 2018) or belonging to a minority group (Gilbey et al., 2020). However, no review has engaged with the full breadth of this literature, resulting in crucial gaps in understanding about how best to prevent mental health problems in children and young people on a population level, and what approaches are most often associated with effective secondary preventative interventions. Our review aims to map this complex field of interventions to provide an evidence overview of promising interventions to reduce child and adolescent mental health problems. In doing so, we aim to provide evidence to inform practice. Our specific review questions are:

- What secondary prevention interventions are effective at reducing mental health problems in children and young people?
- What are the characteristics (such as age, population risk factors and/or symptoms) of young people benefiting from secondary prevention mental health interventions?
- What are the common factors (such as intervention setting, content, or approach) of the effective secondary prevention mental health interventions?

## Methods

The review followed PRISMA guidelines for conducting systematic reviews. The review protocol was registered with PROSPERO (CRD42021290457). The following electronic databases were searched from inception until February 2023, using free text keywords and thesaurus headings: MEDLINE (Ovid); Embase (Ovid); PsycINFO (Ovid); Applied Social Science (ASSIA); Scopus and Cochrane Database of Systematic Reviews (the search strategy is included with the supplementary file). This was supplemented by searching reference lists of relevant studies as well as contacting authors who publish in the field to identify ongoing trials and unpublished work.

Two reviewers independently screened all titles and abstracts using specified inclusion and exclusion criteria, retrieving full papers for all potentially eligible studies, and evaluating in full text. No language or date restrictions were applied. Relevant data were extracted independently by two reviewers, including: study design, sample characteristics, intervention details, outcome measures and intervention effects. Discrepancies at each stage were resolved by discussion or by consulting a third researcher if consensus could not be reached.

### Eligibility

We included systematic reviews of randomised controlled trials, quasi-experimental designs, and outcome evaluations of secondary preventative interventions (either selective or indicated) for children and young people aged 3–17 years or their parents/caregivers. We included reviews of studies outside of this age range providing the mean age of the sample fell within our age range. For reviews of selective intervention, we accepted the authors definition of ‘at risk’ populations. Indicated interventions were required to identify children and young people experiencing mental health distress below the diagnostic threshold with a valid screening tool or assessment. All intervention modalities were considered eligible. Our definition of mental health problems included both internalising (e.g. anxiety and depression) and externalising problems (e.g. aggression, delinquency, behavioural), and self-injury. We excluded reviews of interventions for children and young people with current or historic diagnosed mental health conditions, and reviews of interventions to reduce substance use in children and young people.

### Data Extraction and Synthesis

Data extraction was independently completed by two reviewers using a bespoke, piloted data extraction form. Key characteristics of each of the reviews were recorded, including details of the mental health risk, intervention type (selected or indicated secondary prevention), intervention approach and content, and the main findings of each review. Our primary outcome was the reduction in mental health risk/problem score (assessed using a validated tool or diagnostic interview). Where more than one paper was published from a review, the index review was identified, and additional outcomes of interest reported as linked findings. The findings reported within included reviews were synthesised narratively by intervention type and grouped according to intervention approach and content and presented in tables according to the appraisal of confidence in the evidence (as high/moderate confidence, or low/critically low). This approach to the synthesis is in line with the aims of this systematic review of reviews, resulting in a high-level summary of the evidence (Aromataris et al., 2015).

### Quality Appraisal

We used the Assessment of Multiple Systematic Reviews (AMSTAR 2) tool, a 16-item critical appraisal tool for systematic reviews of healthcare interventions (Shea et al., 2017). The tool assists in the identification of high-quality systematic reviews of randomised and non-randomised studies of interventions and provides an overall rating based on weaknesses in critical domains. The AMSTAR 2 allows for the potential impact of an inadequate rating for each domain and provides a scheme for interpreting critical weaknesses (such as a-priori registration of review protocol, risk of bias assessment of the individual studies) and non-critical weaknesses (such as performing duplication of study selection, duplication of data extraction, reporting of funding sources) within the review. Reviews were not excluded based on quality; rather the AMSTAR 2 was used to assess the confidence in the results of each review (Shea et al., 2017) and our resulting synthesis. Reviews were deemed to provide a high degree of confidence if there is no critical and one non-critical weakness assessed; moderate if there is more than one non-critical weakness; low if there is one critical weakness with or without non-critical weaknesses; critically low if there are more than one critical weakness.

## Results

**Fig. 1:**
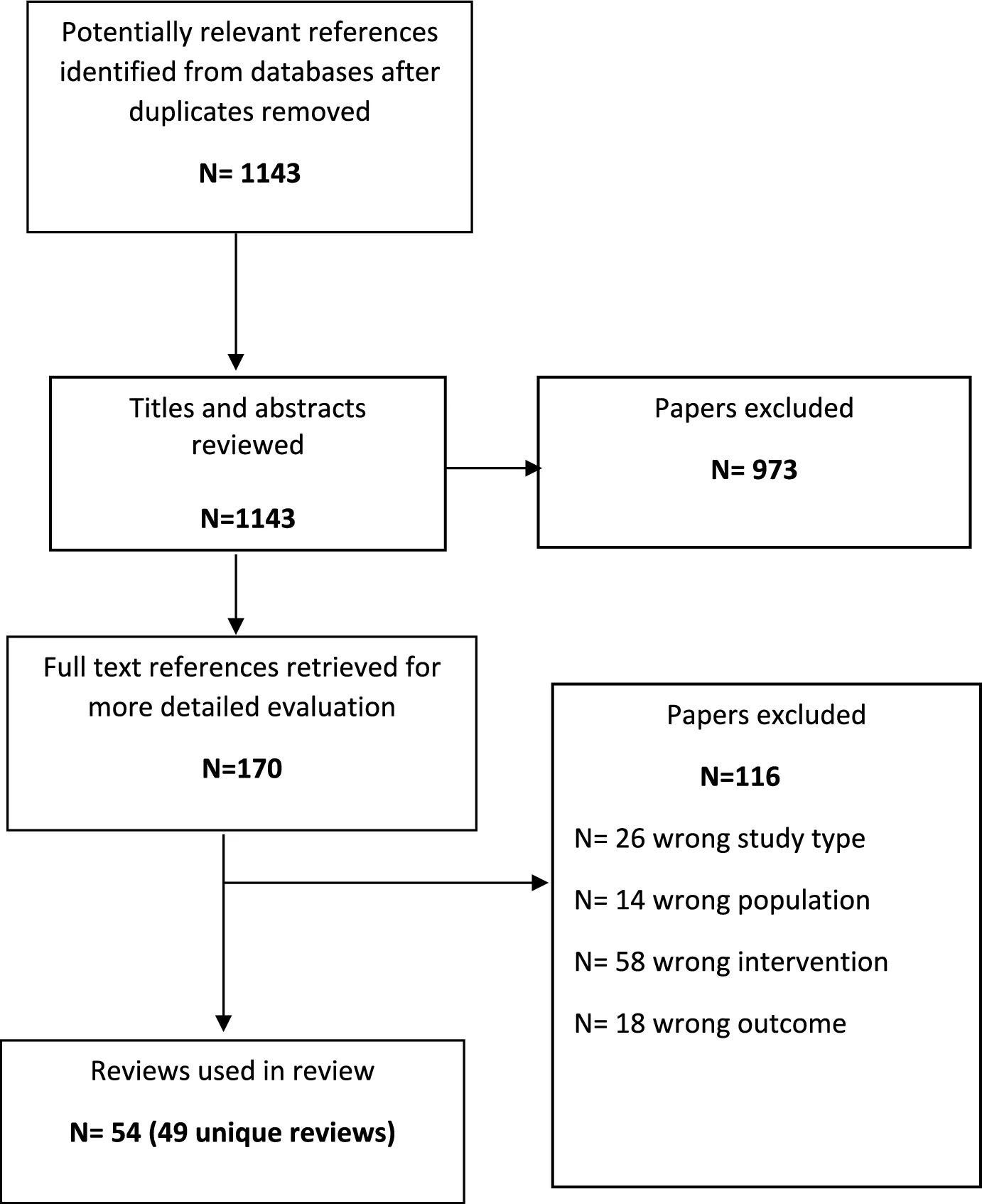
Flow of studies

Our search identified 1433 potentially relevant references. Of those, 170 full papers were retrieved. Fifty-four papers reporting on 49 unique reviews met the inclusion criteria and were included in our systematic review of reviews. Figure 1 shows the flow of studies.

### Description of Studies

The 49 unique reviews each included between 2 and 249 (mean 34) unique studies. Whilst some reviews included a combination of randomised and non-randomised studies, the majority (70%) were reviews of only or mostly randomised controlled trials. The reviews examined selective interventions (n = 22), indicated interventions (n = 15) or a synthesis of both (n = 12). The selective secondary prevention interventions can broadly be grouped into those delivered to children/young people who experienced adversity (Bagneris et al., 2021; Bee et al., 2014; Chen & Panebianco, 2018; Hambrick et al., 2016; Havinga et al., 2021; Lannes et al., 2021; Loechner et al., 2018; Morison et al., 2022; Neil & Christensen, 2009; Papola et al., 2020; Pfefferbaum et al., 2017; Smedler et al., 2015; Thomas et al., 2022; Yohannan et al., 2022), minority groups (Gilbey et al., 2020; Grande et al., 2022; Harlow et al., 2014; Petrenko, 2013), and adolescent mothers (Sangsawang et al., 2019). Reviews examining indicated secondary prevention intervened with children and young people who were identified to be experiencing subclinical internalising problems (anxiety and/or depression) (Baourda et al., 2022; Bertha & Balazs, 2013; Caldwell et al., 2019; Gee et al., 2020; Hetrick et al., 2015; Hugh-Jones et al., 2021; Merry & Spence, 2007; Merry et al., 2011; Rasing et al., 2017; Savaglio et al., 2023; Ssegonja et al., 2018; Thabrew et al., 2018; Werner-Seidler et al., 2017), those that were identified as experiencing subclinical externalising problems (Graaf et al., 2008; Mytton et al., 2002, Mytton et al., 2006; Savaglio et al., 2023), and self-harm (Hawton et al., 2015; Itzhaky et al., 2022; Witt et al., 2021). The confidence in the results of the reviews was rated as high, (n = 12) moderate (n = 5), low (n = 9) and critically low (n = 23). Table 1 provides the critical findings of the reviews which were rated as high or moderate confidence whilst Table 2 provides findings from reviews rated as being of low or very low confidence.

**Table 1:** Reviews with high and moderate confidence findings

Review	Population	Intervention	Comparator	Follow-up	Outcome	Synthesis approach	Confidence rating
Selective intervention							
Adversity							
Bee et al. (2014) 5 studies mixed methods	Children of mental ill parents, aged 2–18 yrs	Combination of therapeutic intervention, psychoeducational and family functioning, parent only, parent child and child only. Focused upon both resilience building and modifying risk	Wait-list control, treatment as usual	Short (6 m), medium (7–12 m)	NS short term CI included a clinically significant reduction in depressive symptoms (ES 0.06; 95% CI –0.20, 0.33)	Meta-analysis	High
Havinga et al. (2021) 10 RCTs	Children of mentally ill parents, mean age 12.3 yrs	CBT, family therapy or skills training, mainly focused on resilience	Wait-list control, information condition, treatment as usual	3–75 m	Reduction in depression/anxiety symptoms at short term (9–18 m) (RR = 0.37; 95% CI 0.21, 0.66) and long term (> 24 months) (RR = 0.71; 95% CI 0.57, 0.87)	Meta-analysis	Moderate
Lannes et al. (2021) 20 RCTs	Children of mentally ill parents, prenatal – age 18 yrs	CBT, psychoeducational and ‘other’, mainly focused upon reducing risk	Wait-list control, attention control, active comparison, treatment as usual	6–12 m	Reduction in the risk of developing any mental health disorder (RR = 0.53; 95% CI 0.34, 0.84, *p* = 0.006)	Meta-analysis	High
Loechner et al. (2018) 14 RCTs	Children of mentally ill parents, mean age 12.8 yrs	CBT and psychoeducational, resilience building	Active and non-active control	Short (5–12 m); long 15–72 m)	Decreases depression incidence (RR = 0.56; 95% CI 0.40, 0.77) but not depressive symptoms (NS)	Meta-analysis	High
Morison et al. (2022) 340 studies mixed methods	Trauma exposed children and adolescents, aged < 18 yrs	Creative arts-based interventions	Wait-list control, treatment as usual, active control	NR	Reduced PTSD symptoms (g = -0.50; 95% CI − 0.78, − 0.23) and negative mood (g = − 0.38 (− 0.64, − 0.11). but NS for externalising difficulties and anxiety	Meta-analysis	Moderate
Papola et al. (2020) 5 RCTs	Children and young people living in low to middle income countries affected by humanitarian crisis, aged 7–18 yrs	Classroom based intervention combining expressive therapy, CBT, psychoeducational, socio-drama, stress inoculation and trauma processing	waitlist, no treatment, treatment as usual, attention control	3 m and study endpoint	NS at reducing PTSD symptoms	Meta-analysis	High
Minority population							
Grande et al. (2022) 2 studies (RCT and quasi)	Indigenous adolescents, aged 12–18 yrs	Resilience enhancing interventions focused on life skills and strengthen communities	Wait-list control, no intervention	> 6 m	Reduced risk factors for suicide	Narrative	Moderate
Indicated							
Internalised problems							
Gee et al. (2020) 45 RCTs	Adolescents with subclinical depression and/or anxiety, aged 10–19 yrs, included trials in low income countries	CBT within school setting, 3–20 session each ranging between 20 and 120 min	Passive control (e.g. waitlist) minimum control (e.g. unguided self-help), active control	Short term (< 6 m) Medium term (> 6- < 12 m), long term (> 12 m)	Reduced depression symptoms with small effect (SMD = 0.34, 95% CI − 0.48, − 0.21) and anxiety with medium effect (SMD = − 0.49, 95% CI − 0.79, − 0.19) in short term. NS medium or long term	Meta-analysis	High
Merry et al. (2011) 39 RCT Merry and Spence (2007), Hetrick et al. (2015)	Children and young people with subclinical depression, aged 5–19 yrs	Mostly CBT and psychoeducational focused upon risk reduction	wait list, no intervention, usual care/health education classes, active intervention	3–9 m, 12 m, 24 m and 36 m	Reduced depression post intervention (RD -0.07; 95% CI − 0.12, − 0.02) and 3–9 m (RD − 0.06; 95% CI − 0.10, − 0.03) with small effect size, NS at 12 m	Narrative	High
Ssegonja et al. (2018) 34 RCTs Rasing et al. (2017)	Children and young people at risk of depression, aged 12–19 yrs	Group CBT within school setting	no intervention, alternative classroom activity)	Up to 13 m	Reduction in incidence (RR 0.43, 95% CI 0.21, 0.87) and symptoms (d − 0.22, 95% CI − 0.32, − 0.11) of depression after 12 m, Reduced anxiety symptoms with a small effect (d = − 0.30; 95% CI [− 0.52, − 0.09]) 3–6 months after the intervention	Meta-analysis	High
Externalising problems							
Mytton et al. (2006) 36 RCTs	Children and young people with aggressive behaviour, aged 4–16 yrs	Primarily school-based resilience intervention, but included parenting skills training and community interventions	No intervention, minimal intervention, treatment as usual, active intervention	12 m	Aggressive behaviour reduced with a small effect size (SMD = − 0.4; 95% CI − 0.73, − 0.06)	Meta-analysis	High
Savaglio et al. (2023) 42 studies Mixed methods	Children exhibiting emotional/behavioural concerns; mean 5.78 yrs	Mostly group programmes delivered to child’s primary caregiver, minority included parent and child components/whole family	Wait-list control, attention control, minimum intervention	NR	Reduction in externalising symptoms (SMD = − 5.6; 95% CI − 0.79, − 0.32), frequency of externalising behaviours (SMD = − 0.54; 95% CI − 0.79, − 0.28) anxiety symptoms (SMD = − 0.25; 95% CI − 0.43, − 0.08) NS anxiety disorder	Meta-analysis	Moderate
Self-harm							
Witt et al. (2021) 17 RCTs Hawton et al. (2015)	Adolescents who have self-harmed, mean age 14.7 yrs	Psychosocial interventions	Treatment as usual, active comparator or combination	Post intervention	Dialectical behaviour therapy significantly reduces repeat self-harm (OR 0.46, 95% CI 0.26, 0.82). NS from other psychosocial approaches	Meta-analysis	High
Combined secondary interventions							
Internalising problems							
Caldwell et al. (2019) 61 RCTs	Children/young people with subclinical depression and/or anxiety, aged 4–18 yrs	Group CBT within school setting, 2–48 sessions	No intervention, waitlist, usual curriculum, attention control	Post intervention, midterm (6-12 m), long term (13–24 m)	NS post intervention and mid-term. Long-term CBT reduced depression (SMD − 0·50; 95% CrI − 0·96, − 0·05) and anxiety (SMD − 0·26, 95% CrI − 0·52, − 0·01) (anxiety outcome based on one study only)	Meta-analysis	High
Externalising problems							
Macarthur et al. (2018) 54 RCTs	Children and young people at risk of antisocial behaviour, aged 9–14 yrs	Individual, family and school level interventions	No intervention, treatment as usual	Up to 12 m	Individual and family level interventions were NS, school-based interventions likely to be effective (OR 0.78, 95% CI 0.59, 1.05; * p* = 0.1)	Meta-analysis	High
Self-harm							
Calear et al. (2016) 29 RCTs	Children and young people at risk of suicide (included universal populations), mean age 15.6 yrs	Social support, CBT, dialectical behavioural therapy, problem solving, motivational interviewing, psychoeducation, mainly focused on risk reduction	no intervention, waitlist, attention control, treatment as usual	Post test-18 m	Half of the secondary prevention studies were found to reduce suicide ideation and/or attempts	Narrative	Moderate
Unspecified mental health problems							
Pilling et al. (2020) 138 RCTs	Children at risk of mental health problems, aged 4–18 yrs	CBT, parenting programmes, psychoeducational or psychotherapeutic, 1–144 sessions	No intervention, wait list, attention control, treatment as usual, active intervention	12 m	Reduced overall mental health problems with small to medium effect size (g = 0.31, 95% CI 0.25–0.37)	Meta-analysis	High

**Table 2:** Reviews with low and critically low confidence findings

Review	Population	Intervention	Comparator	Follow-up	Outcome	Synthesis approach	Confidence rating
Selective interventions							
Adversity							
Bagneris et al. (2021) 21 RCTs	Trauma exposed children, aged 5–11 yrs, school setting	Psychoeducational; includes risk reduction and resilience building, parent and child, mostly focused on skill enhancement	Wait-list control, active comparator	6–30 m	Reduced posttraumatic stress symptoms	Meta-analysis	Critically low
Chen and Panebianco (2018) 9 studies mixed methods	Bereaved children, aged 3–5 yrs	Play therapy, expressive arts therapy, family therapy, and CBT	Control group	NR	Expressive arts only reduced behavioural problems with small effect size	Narrative	Critically low
Hambrick et al. (2016) 39 RCTS & quasi experimental	Children in foster care, aged 0–12 yrs	CBT, family therapy, foster care skills training and psychological interventions mostly focused on skill enhancement	Treatment as usual	3–24 m	All interventions reported improvements in mental health	Narrative	Low
Harlow et al. (2014) 3 studies mixed methods	Indigenous youth	Multi-component suicide prevention programmes,	NR	NR	Mixed results at preventing suicide	Narrative	Low
Neil and Christensen (2009) 3 RCTs	Children who have experienced parental divorce	CBT, psychoeducation, relaxation and modelling	Wait-list control, no intervention, attention control	1–12 m	Two out of three trials were found to reduce anxiety	Narrative	Critically low
Pfefferbaum et al. (2017) 11 RCTs	Children who have experienced disaster, 0–18 yrs	CBT, narrative exposure, meditation relaxation, debriefing, eye movement desensitization and reprocessing	Wait-list control, attention control, active comparator, no treatment control	Post intervention to 24 m	NS on reducing posttraumatic stress symptoms	Narrative	Critically low
Smedler et al. (2015) 36 RCTs & 2 quasi experimental	Families affected by internal stress/separation and divorce, aged 2–19 yrs	Parentings programmes, focused on reducing risk	No intervention control, treatment as usual	6 m >	Mixed results for reduction in child behaviour	Meta-analysis	Critically low
Thomas et al. (2022) 11 studies Mixed methods	Children living in conflict-affected areas, low-and-middle income countries, aged < 18 yrs	Trauma-focused CBT (TF-CBT)	Waitlist control, usual care	4–12 m	TF-CBT was effective at treating trauma-related symptoms	Narrative	Critically low
Wang et al. (2020) 12 RCTs	Left behind children from China, aged < 18 yrs, low-middle income country	Psychological therapy, psychoeducational, mostly focused on resilience	NR	Post intervention	Most studies reported improvements in mental health	Narrative	Critically low
Watters and O'Callaghan (2016) 5 studies mixed methods	Children and adolescents in street situations, aged 12–25 yrs	Residential programme, multidisciplinary (MD) approach, resilience training, psychodrama, narrative exposure therapy	NR	3–5 yr	MD approach, resilience training and psychodrama improved mental health, narrative exposure therapy increased PSTD	Narrative	Critically low
Yohannan et al. (2022) 94 studies Mixed methods	Trauma exposed children; aged 3-18 yrs	CBT	NR	NR	CBT reduces PTSD	Meta-analysis	Critically low
Minoritised groups							
Gilbey et al. (2020) 5 studies mixed methods	LGBTQ + young people, aged 12–25 yrs	Digital health interventions	NR	NR	NS	Narrative	Low
Petrenko (2013) 17 RCTs and quasi experimental	Children with developmental disabilities, aged 3–8 yrs	Parenting programmes, focused on reducing risk	Wait-list control, treatment as usual	3–24 m (median 6 m)	15/17 (88%) studies reported significant reductions in child behaviour problems. Mostly moderate to large effect sizes	Narrative	Critically low
Other vulnerable attributes							
Sangsawang et al. (2019) 13 RCTs	Adolescent mothers, aged 12–19 yrs	Home visiting, prenatal antenatal/postnatal parenting programmes, CBT and psychoeducational approaches, social support interventions, early intervention programs (EIP), infant massage training	Care as usual, optimised standard care, educational intervention	6 weeks–36 m post-partum	Postpartum depression symptoms reduced pre and postnatal educational programs, psychoeducational and CBT, interpersonal therapy infant massage training. Mixed findings for home visiting, NS social support	Narrative	Critically low
Indicated interventions							
Internalising problems							
Bertha and Balazs (2013) 6 studies mixed methods	Adolescents with subclinical depression, aged 10–19 yrs	Group CBT, expressive therapy, interpersonal therapy, internet based intervention	No treatment control, attention control	3–24 m	Group CBT and internet based programmes reduced later major depressive episode but not expressive or interpersonal therapy	Narrative	Critically low
Baourda et al. (2022) 12 controlled trials	Children & adolescents with anxiety symptoms, aged 5–17 yrs	Group psychoeducation	Wait-list control, active control	NR	Reduction in anxiety symptoms with moderate effect size	Meta-analysis	Low
Hugh-Jones et al. (2021) 20 RCTs	Children and young people with subclinical anxiety, aged 5–18 yrs	Group CBT within school setting	Wait-list control, no intervention, attention control	< 6 m, 6–12 m, 12 m, 24 m	Anxiety disorder reduced at 12 m and 24 m	Meta-analysis	Critically low
Neil and Christensen (2009) 3 RCTs	Children and young people with subclinical anxiety, aged 8–17 yrs	CBT, psychoeducation, relaxation and modelling	Wait-list control, no intervention, attention control	1–12 m	Two out of three trials were found to reduce anxiety	Narrative	Critically low
Thabrew et al. (2018) 5 RCTs	Children and young people with disabilities who have subclinical depression and anxiety, aged 10–18 yrs	e-Health interventions based upon CBT and psychoeducation, mostly focused upon risk reduction	Attention control, treatment as usual, educational intervention	Post intervention and 3–6 m	NS for reduction in depression and anxiety	Meta-analysis	Low
Werner-Seidler et al. (2017) 81 RCTs	Children and young people with subclinical depression and anxiety, aged 5–19 yrs	Individual and group CBT, interpersonal therapy and psychoeducational programme	No intervention, school as usual, wait-list control, attention control, alternate educational/psychological condition	Post intervention, short term (0–6 m), medium (6–12 m), long (> 12 m)	Depression and anxiety reduced at 12 m with a small effect size	Meta-analysis	Critically low
Externalising problems							
Park-Higgerson et al. (2008) 19 RCTs	Violent youth, aged 6–17 yrs, school setting	CBT and psychoeducational, mainly focused on reducing risk	Control	1–12 m	Reduced aggressive behaviour	Meta-analysis	Critically low
De Graaf et al. (2008) 14 RCTs & 1 quasi experimental	Children with behaviour problems, aged 2–12 yrs	Parenting programme	Wait-list control, no treatment, usual care, standard Triple P	6–12 m	Reduced child behaviour problems with moderate to large effect size	Meta-analysis	Low
Itzhaky et al. (2022) 25 RCTs	Adolescents who have self-harmed, aged 10–19 yrs	Structured, psychosocial intervention	No intervention, usual care, enhanced usual care, active comparison	6 m follow-up (or closest time point)	Suicide ideation reduced with small effect; NS on suicide attempt or self-harming behaviour	Meta-analysis	Low
Smedler et al. (2015) 36 RCTs & 2 quasi experimental	Children and young people with subclinical externalising problems, aged 2–19 yrs	Parentings programmes, focused on reducing risk, weekly sessions for 3–9 m	No intervention, treatment as usual	6 m >	NS for reduction in child behaviour	Meta-analysis	Critically low
Combined secondary interventions							
Internalising problems							
Christensen et al. (2010) 44 trials	Youth at risk of anxiety and depression, aged 11–25 yrs	CBT, exercise, stress management or other	No intervention, wait-list control, attention control	2–36 m	CBT consistently found to reduce anxiety and depression	Narrative	Low
Cuijpers et al. (2006) 8 RCTs	Children and young people at risk of depression, aged 7–19 yrs	CBT within school setting, 8–16 sessions	Usual care, waiting list, no treatment control, attention placebo	Post intervention	Reduced depression with moderate effect size	Meta-analysis	Critically low
Horowitz and Garber (2006) 18 RCTs	Children and young people at risk of depression, aged 7–25 yrs	Combines range of selective and indicated interventions	NR	3–8 m	Both selective and indicated interventions were more effective at reducing depression than universal interventions	Meta-analysis	Critically low
Schwartz et al. (2019) 8 RCTs	Children and young people at risk of anxiety, aged < 18 yrs, high income countries, includes universal populations	Mostly CBT-based and psychoeducational interventions, 2 non-CBT interventions used computer delivery	NR	9 m–3.75 yrs	Reduced anxiety	Meta-analysis	Low
Externalising problems							
De Vries et al. (2015) 39 trials	Children and young people at risk of delinquency, mean age 14 yrs	CBT, behaviour modification, interpersonal problem solving, social skills training, life skills training, anger management, moral reasoning, meditation, mentoring, delivered to young people and/or parents, mostly focused on risk reduction	Treatment as usual, no treatment, minimal contact, wait-list, alternative treatment, placebo	NR	Reduced delinquency with a small effect size. Behavioural-orientated programmes focused upon parenting skills yielded largest effects	Meta-analysis	Critically low
Wilson and Lipsey (2007) 249 trials Wilson et al. (2003)	Children and young people at risk of aggression, preschool children to high school	School-based interventions delivered outside of classroom	NR	Post intervention	Reduced aggression with small effect size	Meta-analysis	Critically low
Unspecified mental health problems							
Waddell et al. (2007) 15 RCTs	Children and young people at risk of mental disorders, 0–18 yrs	Group CBT, group therapy, group social skills, individual parent training	Wait-list, usual care, no intervention	Post intervention-12 m	All interventions reduced conduct disorder, CBT reduced depression, NS anxiety	Narrative	Critically low
van Genugten et al. (2017) 40 trials	Young people at risk of mental health problems, aged 12–18 yrs	CBT and social skills training, half interventions school-based, mostly delivered by health professional, average number of sessions was 16.2	NR	Short (post intervention-6 m), long (6 > m)	Reduced internalising problems	Meta-analysis	Critically low

### Selective Interventions

Reviews providing high to moderate confidence evidence for selective interventions were mostly delivered to groups of children and young people who had experienced adverse childhood experiences; most of which found evidence of effectiveness. This adversity in the majority of reviews related to having a parent who experienced mental health problems. Reviews typically examined a range of interventions and combined these within their estimates of effect. As each included intervention may vary in effectiveness, it was difficult to confidentially determine which of the interventions were effective. However, reviews reporting effective interventions typically included cognitive behavioural therapy (Havinga et al., 2021; Lannes et al., 2021; Loechner et al., 2018) or psychoeducation approaches (Bee et al., 2014; Lannes et al., 2021; Loechner et al., 2018) focused upon building resilience (Bee et al., 2014; Havinga et al., 2021; Loechner et al., 2018), with some reviews also including creative arts-based interventions (Morison et al., 2022), family therapy and skills training (Bee et al., 2014; Havinga et al., 2021). Children/young people whose parents experienced mental health problems were found to have a 47% reduction in developing the same mental health disorder as their parents at 9–24 months follow-up (Lannes et al., 2021). Significant reductions in depressive symptoms (Loechner et al., 2018) and incidence of internalising disorders (Lannes et al., 2021) were also reported by reviews examining secondary prevention in this population, with reviews reporting between 44% (Loechner et al., 2018) and 63% reduction (Havinga et al., 2021) at 12–15 month follow-up and 29% reduction at 24 month follow-up (Havinga et al., 2021). Whilst a review of creative arts-based interventions for children and adolescents exposed to traumatic events reported significant reduction of PTSD symptom scores and negative mood post-intervention compared to control group, however reductions were not found for externalising problems or anxiety (Morison et al., 2022). Only one review examining secondary prevention for children who experienced adversity did not report reductions in mental health problems. This review found there may be no evidence of a difference between psychological and social intervention groups and control groups for reducing PTSD symptoms, depressive symptoms and anxiety symptoms in children aged 7–18 years affected by humanitarian crisis at study endpoint (Papola et al., 2020).

We identified one review contributing moderate certainty evidence examining interventions for suicide prevention within indigenous adolescents. This review found only two studies meeting the inclusion criteria. Both of these studies showed evidence of effectiveness as reducing risk factors and increasing protective factors associated with suicide, however the effect upon suicide ideation or attempts was not assessed (Grande et al., 2022).

Further reviews reporting evidence of low to critically low confidence reported mixed evidence. These reviews could broadly be categorised as those examining selective interventions for children and young people who had experienced adversity and those from minoritised groups. Reviews of selective interventions for groups who had experienced adversity were mostly found to be effective, including resilience enhancing interventions (Hambrick et al., 2016; Sangsawang et al., 2019; Wang et al., 2020; Watters & O'Callaghan, 2016); parenting skills training (Smedler et al., 2015), CBT (Yohannan et al., 2022). Only one review reported the potential for an increase in anxiety following a debriefing intervention for young people who had been exposed to a disaster (Pfefferbaum et al., 2017). Conversely, interventions for minoritised youth were mostly found not to be effective at reducing mental health problems in their samples; one being a sexual minoritised population (Gilbey et al., 2020), and another being an ethnic minority population (Harlow et al., 2014). One narrative synthesis examining parenting interventions for children with developmental disabilities found that interventions were effective at reducing mental health problems in children (Petrenko, 2013). A further review reported school-based interventions were effective at reducing violence in at-risk young people (Park-Higgerson et al., 2008).

### Indicated Interventions

Reviews which provided high confidence evidence on indicated interventions mostly found interventions to be effective at reducing externalising problems in children and young people. Interventions which aim to enhance resilience through developing relational and social skills delivered directly to children and young people within the school setting, were found to be effective at reducing aggressive and violent behaviour with a small effect size at post intervention (Mytton et al., 2002; Mytton et al., 2006), with similar effects found at 12 month follow-up (Mytton et al., 2002; Mytton et al., 2006). A further review which mostly included resilience enhancing parenting interventions reported reductions in externalising symptoms and frequency of externalising behaviours with a moderate effect size in young children (Savaglio et al., 2023).

Interventions for children and young people with subclinical internalising problems mostly consisted of CBT focused upon modifying risk, and were often effective at reducing depression symptoms post intervention and/or at short term follow-up (usually 6 months) (Gee et al., 2020; Rasing et al., 2017; Ssegonja et al., 2018), and long term follow-up (12 months) (Rasing et al., 2017; Ssegonja et al., 2018). Evidence of effect upon anxiety symptoms was limited to post intervention (Gee et al., 2020), and short term follow-up (Rasing et al., 2017). A review of mostly resilience enhancing parenting interventions reported reductions in anxiety symptoms but not disorder (Savaglio et al., 2023).

A further review examining psychotherapy for self-harm in children found evidence that dialectical behaviour therapy reduces repetition of self-harm at post-intervention however the review reported there was no evidence for other psychosocial approaches (Witt et al., 2021).

Reviews contributing low and critically low confidence evidence examined a range of indicated interventions, with limited evidence of effect. There were mixed results for the effectiveness of parenting interventions to reduce child behaviour problems (Graaf et al., 2008; Smedler et al., 2015). Meta-analyses found indicated interventions to be effective at reducing depression (Merry & Spence, 2007; Merry et al., 2011; Werner-Seidler et al., 2017) and anxiety symptoms (Baourda et al., 2022; Hugh-Jones et al., 2021) whilst narrative reviews reported inconsistent effects upon symptoms of depression (Thabrew et al., 2018) and anxiety (Neil & Christensen, 2009). A meta-analysis reported that psychosocial interventions reduced suicide ideation with a small effect, but not suicide attempts or self-harming behaviour (Itzhaky et al., 2022).

### Combined Secondary Interventions

Four reviews contributing high or moderate confidence evidence examined a combination of selective and indicated interventions. These reviews typically found evidence of effect. A review which examined the effectiveness of secondary prevention to reduce a range of mental health problems found that interventions were effective at preventing conduct disorder, depression, anxiety and PTSD at post intervention and 12 month follow-up and that there was some evidence to suggest that younger children (under the age of 12 years) may experience greater benefit than older children (Pilling et al., 2020). A review reported evidence that CBT reduced depression symptoms compared to wait-list control with a moderate effect size in primary school children but not secondary school children and reduced anxiety only in secondary school children at 13–24 months follow-up (Caldwell et al., 2019). However, it should be noted that this evidence was based upon the findings of a single study. A review of individual, family and school-level interventions found secondary prevention within schools were effective at reducing anti-social behaviour at 6 months follow-up (Macarthur et al., 2018). Whilst a further review of the effectiveness of school-based interventions to reduce suicidality reported mixed results with around half of the included studies finding no effect (Calear et al., 2016).

Reviews contributing low and critically low confidence evidence mostly reported one or more intervention which were found to be effective at reducing mental health problems in children and young people. This included an effect upon externalising problems only (Vries et al., 2015; Wilson & Lipsey, 2007; Wilson et al., 2003), internalising problems only (Christensen et al., 2010; Cuijpers et al., 2006; Horowitz & Garber, 2006; Schwartz et al., 2019) and a range of externalising and internalising problems (Genugten et al., 2017; Waddell et al., 2007). They mostly reported small effects from the preventative interventions, with the exception of one review which estimated a reduction in anxiety with a large effect size following the Coping and Promoting Strength intervention (Schwartz et al., 2019). Due to these reviews examining very different secondary interventions, in different populations, it is difficult to draw further conclusions from the overview of the combined preventative interventions.

## Discussion

Our systematic review of reviews has found a large body of evidence to support both selective and indicated interventions in a range of populations and settings. This builds upon an established evidence for preventative interventions in children and young people at risk of developing mental health problems (World Health Organization, 2020). However, the largest high and moderate confidence evidence was for selective interventions with children and young people who experienced adversity. Research consistently demonstrates that our early life experiences are of substantial importance to our health throughout the life course (Britto et al., 2017; Marmot et al., 2010). Where those early experiences are adverse, there is an immediate and long-lasting impact upon children (Bellis et al., 2015), resulting in major health and financial burden globally (Hughes et al., 2021). As such, our finding that selective preventative interventions with this group maybe effective at preventing mental health problems has important implications for practice.

The children and young people targeted by the selective interventions included those that had experienced a range of adverse childhood events, however the most common adversity related to parental mental health problems. Recent research highlighted the association between parental and child parental mental health problems, particularly in populations impacted by other family adversity such as poverty (Adjei, 2022). Whilst there is a large evidence base for interventions to address parental mental health problems, most are examining the effectiveness of interventions provided to the parent (Barrett et al., 2023). Whilst these approaches can be effective in reducing parental mental health problems, it is likely that children and young people will also require intervention to help them to overcome the impact of exposure to this adversity (McGovern et al., 2021). Whilst we found evidence that interventions were effective for children and young people who are experiencing a range of mental health problems, there was limited evidence for PTSD and self-harm. Positive effect was noted most often for those children and young people who experienced common internalising or externalising problems. Given the high prevalence of these mental health difficulties in children and young people who experience adversity (Benjet et al., 2010; Hughes et al., 2017; Parkes, 2020), our findings highlight an opportunity to affect change in this vulnerable group of children and young people, contributing to national (Wykes et al., 2023) and global (United Nations, 2015) efforts to address the disease burden caused by mental ill health and promote future health and wellbeing.

Across the body of reviews, we found that CBT, psychoeducation and family therapy and skills training were often included in reviews which reported positive outcomes. Effective interventions included those provided at an individual or family level. The components of these approaches varied but could be broadly categorised as having content focused upon reducing risk or enhancing resilience, with evidence supporting both of these approaches. Risk reduction interventions typically consist of components which may bring about a change in behaviours and thoughts which contribute to symptoms (Wampold, 2015), whilst interventions which aim to enhance resilience tend to include the development of personal resources and reinforcement of protective factors (Reavley, 2015). As many of the included reviews combined multiple studies of diverse interventions, we were unable to identify specific intervention content that is most likely to be associated with improvements in mental health beyond these broad categories. It is possible that a combination of components focused upon risk reduction and resilience enhancement across common behavioural, interpersonal, cognitive, and emotional domains maybe beneficial to improving mental health outcomes in children and young people (Singla et al., 2017; Skeen et al., 2019). Research has demonstrated the mediating effect that parent–child conflict has upon the development of mental health problems in children (Healy et al., 2022; Burt et al., 2003), with up to half of all externalising difficulties and one fifth of internalising difficulties in children who have experienced adversity being explained by parent–child conflict (Dhondt et al., 2019). Given the evidence for family level interventions highlighted within our review, a selective preventative intervention which includes components focused upon the parent–child interaction may be beneficial, although further research is needed to examine effectiveness.

Taken together, the findings of our review of reviews have important implications for practice. They provide support for early preventative intervention with children who have experienced adversity before they develop mental health concerns. This selective approach negates individual identification of risk which can be challenging and costly to implement (Dodge, 2020). It is likely that the selective intervention should contain both risk reduction and resilience enhancing components provided to both individual young people and their families.

### Strengths and Limitations

Our review of reviews engages with a large international literature covering a wide range of secondary preventative interventions for the breadth of child and adolescent mental health problems. A strength of this approach is that it provides an accessible overview of up-to-date evidence covering multiple sub-populations of young people in a range of contexts. Further, our fields of enquiry can be separately considered with the potential to inform practice in this area (Pollock et al., 2020). Through rigorous methods, including duplicate screening and data extraction, we have been able to highlight promising interventions and situations to promote the mental health of children and young people. However, our review is not without its limitations. A proportion of the reviews report on the same individual studies. Within the high and moderate confidence evidence there are n = 40 studies which are included in more than one review (n = 34 included in 2 reviews; n = 5 included in three and n = 1 included in four reviews). Whilst this is not unusual in a review of reviews of this size, it does result in additional weight being given to some of the evidence. Many of the included reviews also took a broad approach when reviewing the literature, often including studies with participants from a wide age range and did not provide details of the mean age of participants. Consequently, the actual age of the children and young people who received the intervention was often unclear and therefore we were unable to identify the optimum age at which to intervene to prevent mental health problems developing. Reviews all included a range of outcome measures including self-report, validated screening tools and, to a lesser extent, diagnostic interviews. As such, the original studies which are included within the reviews may be at risk of bias. Reviews typically combined different secondary preventative interventions and reported upon pooled effects (e.g. producing of meta-analysis of CBT, psychoeducation and family therapy approaches) limiting our ability to confidentially determine which intervention types were most often found to be effective. The intensity and duration of these interventions varied greatly and as such it is unclear what intervention dose is effective. Further, comparison conditions within reviews often varied from no intervention control to an active comparison intervention, resulting in inflated and deflated results. Only a minority of the included reviews examined interventions trialled within low-middle income countries. As such, the findings of this evidence overview may not generalise to these countries.

## Conclusion

Interventions to prevent mental health problems in young people are more likely to be effective if they combine both risk reduction and resilience enhancing approaches to targeting younger children and young people who have experience adversity and/or those with subclinical externalising and internalising difficulties. Interventions may include both individual and family level interventions components.

## Supplementary Information

Below is the link to the electronic supplementary material.

Supplementary file1 (DOCX 18 KB)
