# Supplementary material for: The Effectiveness of Preventative Interventions to Reduce Mental Health Problems in at-risk Children and Young People: A Systematic Review of Reviews
Source: J Prev (2022). 2024 Jun 17;45(4):651–84. doi: 10.1007/s10935-024-00785-z (PMC11271346; doi:10.1007/s10935-024-00785-z)
Supplement: Supplementary file 1 — Supplementary file1 (DOCX 18 KB) [file 10935_2024_785_MOESM1_ESM.docx]

**MEDLINE OVID search strategy**

1. Child* OR young person OR young people OR adolescen* OR pre*school* OR preschool*
2. child/
3. 1 OR 2
4. Mental health OR wellbeing OR resilien* OR conduct OR depress* OR anxiety OR suicide OR self*harm OR internali* OR externali* OR trauma OR aggressi* OR disruptive OR behavio* problem* OR delinquent* OR anti*social
5. *mental health/
6. 4 OR 5
7. Prevent* adj3 risk
8. Secondary adj3 prevent*
9. secondary ADJ3 intervention*
10. secondary adj3 indicated
11. indicated ADJ3 intervention*
12. select* adj3 intervention*
13. select* adj3 prevent*
14. risk adj3 intervention*
15. prevent* adj3 program*
16. Secondary prevention/exp
17. 7 OR 8 OR 9 OR 10 0R 11 OR 12 OR 13 OR 14 OR 15 OR 16
18. meta*analysis OR evidence review OR evidence assessment
19. Systematic adj3 review
20. 13 OR 14
21. 3 AND 6 AND 17 AND 20

**EMBASE OVID search strategy**

1. Child* or young person or young people or adolescen* or pre*school* or preschool*).ab,ti
2. exp Child Psychiatry/ or exp Child Psychology/ or exp Child Health/
3. (Mental health or wellbeing or resilien* or conduct or depress* or anxiety or suicide or self*harm or internali* or externali* or trauma or aggressi* or disruptive or behavio* problem* or delinquent* or anti*social).ab,ti
4. exp Mental Health/
5. (Prevent* adj3 risk).ab,ti
6. (Secondary adj3 prevent*).ab,ti
7. (secondary adj3 intervention).ab,ti.
8. (secondary adj3 indicated).ab,ti.
9. (indicated adj3 intervention).ab,ti.
10. (select* adj3 intervention).ab,ti.
11. (select* adj3 prevent*).ab,ti.
12. (risk adj3 intervention*).ab,ti.
13. (prevent* adj3 program*).ab,ti.
14. exp preventive medicine/ or exp preventive health services/ or exp early intervention/
15. (meta*analysis or evidence review or evidence assessment).ab,ti.
16. (systematic adj3 review).ab,ti.
17. 1 or 2
18. 3 or 4
19. 5 or 6 or 7 or 8 or 9 or 10 or 11 or 12 or 13 or 14
20. 15 or 16
21. 17 and 18 and 19 and 20

**PsycInfo OVID search strategy**

1. Child* or young person or young people or adolescen* or pre*school* or preschool*).ab,ti
2. exp Child Psychiatry/ or exp Child Psychology/ or exp Child Health/
3. (Mental health or wellbeing or resilien* or conduct or depress* or anxiety or suicide or self*harm or internali* or externali* or trauma or aggressi* or disruptive or behavio* problem* or delinquent* or anti*social).ab,ti
4. exp Mental Health/
5. (Prevent* adj3 risk).ab,ti
6. (Secondary adj3 prevent*).ab,ti
7. (secondary adj3 intervention).ab,ti.
8. (secondary adj3 indicated).ab,ti.
9. (indicated adj3 intervention).ab,ti.
10. (select* adj3 intervention).ab,ti.
11. (select* adj3 prevent*).ab,ti.
12. (risk adj3 intervention*).ab,ti.
13. (prevent* adj3 program*).ab,ti.
14. prevention/ or exp preventive health behavior/ or exp preventive health services/ or exp preventive mental health services/ or exp preventive mental health services/ or exp early intervention/
15. (meta*analysis or evidence review or evidence assessment).ab,ti.
16. (systematic adj3 review).ab,ti.
17. 1 or 2
18. 3 or 4
19. 5 or 6 or 7 or 8 or 9 or 10 or 11 or 12 or 13 or 14
20. 15 or 16
21. 17 and 18 and 19 and 20

**Proquest Applied Social Sciences Index & Abstracts (ASSIA) search strategy**

[(MAINSUBJECT.EXACT.EXPLODE("Childhood") OR ab(Child* OR young person OR young people OR adolescen* OR pre*school* OR preschool*)) AND (MAINSUBJECT.EXACT.EXPLODE("Mental health") OR ab(Mental health OR wellbeing OR resilien* OR conduct OR depress* OR anxiety OR suicide OR self*harm OR internali* OR externali* OR trauma OR aggressi* OR disruptive OR behavio* problem* OR delinquent* OR anti*social)) AND ((MAINSUBJECT.EXACT("Mental health promotion") OR MAINSUBJECT.EXACT("Mental health services")) OR ab(Prevent* NEAR/3 risk) OR ab(Secondary NEAR/3 prevent*) OR ab(secondary NEAR/3 intervention*) OR ab(secondary NEAR/3 indicated) OR ab(indicated NEAR/3 intervention*) OR ab(select* NEAR/3 intervention*) OR ab(select* NEAR/3 prevent*) OR ab(risk NEAR/3 intervention*) OR ab(prevent* NEAR/3 program*)) AND (ab(meta*analysis OR evidence review OR evidence assessment) OR MAINSUBJECT.EXACT.EXPLODE("Systematic reviews") OR ab(Systematic NEAR/3 review))](https://www.proquest.com/myresearch/savedsearches.checkdbssearchlink:rerunsearch/2045006/SavedSearches?t:ac=SavedSearches)

**Scopus search strategy**

1. Mental health OR wellbeing OR resilien* OR conduct OR depress* OR anxiety OR suicide OR self*harm OR internali* OR externali* OR trauma OR aggressi* OR disruptive OR behavio* problem* OR delinquent* OR anti*social
2. Child* OR young person OR young people OR adolescen* OR pre*school* OR preschool*
3. Prevent* W/3 risk
4. Secondary W/3 prevent*
5. secondary W/3 intervention*
6. indicated W/3 intervention*
7. select* W/3 intervention*
8. select* W/3 prevent*
9. prevent* W/3 program*
10. 3 OR 4 OR 5 OR 6 OR 7 OR 8 OR 9
11. meta*analysis OR evidence review OR evidence assessment OR systematic review
12. 1 AND 2 AND 10 AND 11
